# Thyroid hormone determines energy efficiency of locomotion in zebrafish (*Danio rerio*) in a temperature-sensitive manner

**DOI:** 10.1242/jeb.252605

**Published:** 2026-07-16

**Authors:** Miki Jahn, Frank Seebacher

**Affiliations:** School of Life and Environmental Sciences, University of Sydney, Sydney, NSW 2006, Australia

**Keywords:** Metabolism, Swimming performance, Acclimation, Kinematics, Fineness ratio

## Abstract

The energetic cost of animal movement has important ecological ramifications because it can comprise a large proportion of energy budgets. Understanding the mechanisms that determine energetic efficiency of locomotion (cost of transport; ∫CoT) is important to estimate environmentally induced changes in movement patterns and energy allocation. We aimed to determine whether thyroid hormone and differences in morphology underlie individual differences in ∫CoT in zebrafish (*Danio rerio*). We found that pharmacologically induced hypothyroidism increased ∫CoT particularly at cooler temperatures (24°C cf. 30°C). Hypothyroid fish also had a lower critical swimming speed (*U*_crit_) and a reduced tailbeat amplitude overall, but increased amplitude at higher swimming speed. Tailbeat frequency increased with increasing speed, but hypothyroidism modulated these responses. Fineness ratio and cross-sectional area were unrelated to ∫CoT. Our data indicate that ∫CoT is embedded within the broader thyroid regulatory network, which renders efficiency of movement sensitive to environmental temperature changes and endocrine-disrupting pollution.

## Teaser

**Summary:** Environmental temperatures and thyroid hormone influence energy allocated to movement. Climate change can disrupt these processes and thereby influence ecology.

## INTRODUCTION

Locomotion underpins fitness-related behaviours such as foraging, predation, dispersal and reproduction (Cespedes and Lailvaux, 2015; Domenici et al., 2019; Wilson et al., 2015). Energy invested in locomotion reduces the energy available for other physiological processes, including growth and reproduction (Pontzer and McGrosky, 2022), and differences in locomotor efficiency therefore shape individual energy budgets (Labonte and Holt, 2022). The energetic cost of locomotion can be estimated as the cost of transport (CoT), and is measured as the mass-specific aerobic energy used for a given distance travelled at different speeds (Claireaux et al., 2006). As animals regularly travel at a range of speeds, integrating CoT (∫CoT) across ecologically relevant speeds provides a measure for overall locomotor efficiency in individuals (Seebacher et al., 2016). ∫CoT varies among individuals within species, but it is a repeatable trait within individuals in zebrafish (*Danio rerio*) (Jahn and Seebacher, 2019). Differences between individuals in ∫CoT mean that conspecifics of the same population need to allocate different amounts of energy to the same locomotor task, and may therefore experience different energy allocation challenges within the same environmental or social contexts (Killen et al., 2017). Increasing temperature also increases ∫CoT and differences between individuals, thereby exacerbating differences in energy allocation strategies and behaviour (Pilakouta et al., 2023; Seebacher and Krause, 2017). Identifying the mechanisms underlying individual variation in ∫CoT is therefore important to predict animal performance and behaviour in changing environments, which may ultimately influence biogeography (Wrensford et al., 2025).

Thyroid hormone (TH) is a potential regulator of ∫CoT as it is a metabolic regulator that influences growth, muscle function and morphology in fish and other vertebrates (Deal and Volkoff, 2020; Zwahlen et al., 2024). In skeletal muscle, TH regulates myosin heavy chain expression, shifting muscle towards fast-twitch phenotypes that have greater contractile capacities (Salvatore et al., 2014). Additionally, muscle contraction and relaxation depend largely on Ca^2+^ release from the sarcoplasmic reticulum and re-sequestration by the sarco-endoplasmic reticulum ATPase (SERCA) (Gundersen, 2011). TH increases overall SERCA expression, leading to increased rates of relaxation (Salvatore et al., 2014). Overall, TH can therefore increase contractile speed and potentially swimming performance and tail beat amplitude and frequency (Little, 2021; Seebacher and Walter, 2012). In endotherms, these shifts in muscle phenotype can be associated with decreased energetic efficiency of SERCA to facilitate heat production, but this is not generally the case for ectotherms (Gamu et al., 2020; Nowack et al., 2017). However, TH may influence the efficiency of locomotion in ectotherms by regulating muscle phenotypes, metabolism and cardiovascular function in a temperature-sensitive manner (Little, 2021). Disruption of TH signalling can decrease SERCA activity, metabolic rate and heart rate and thereby constrain locomotion (Little, 2021), and possibly shift proximate ATP production to less efficient anaerobic pathways because of restricted oxygen delivery.

Notably, TH actions are sensitive to temperature and TH is particularly important for thermal acclimation in fish, so it may be expected that the actions of TH interact with temperature (Little et al., 2013; Zak et al., 2017). Interestingly, the thermal sensitivity of TH actions can vary between species, and TH was shown to mediate cold acclimation in zebrafish (Little et al., 2013), but was more important in facilitating warm acclimation in lake whitefish (*Coregonus clupeaformis*) (Zak et al., 2017).

In addition to physiological responses, morphological variation among individuals may influence CoT and swimming performance by altering hydrodynamic drag and propulsion (Liao, 2022; Sun et al., 2025). In fish, body shape is often quantified using the fineness ratio (length/maximum width), where a greater fineness ratio indicates more streamlined morphologies that exhibit lower CoT and improved swimming performance (Conradsen et al., 2016; Ohlberger et al., 2006). Fish with higher fineness ratios also had reduced tailbeat frequencies and oxygen consumption for a given length compared with less streamlined morphotypes (Blake et al., 2009). Interestingly, morphological differences can influence swimming performance in a temperature-dependent manner in zebrafish (Miller et al., 2025), and potentially morphology, thyroid signalling and temperature can have interactive effects on ∫CoT.

Our aim was to determine whether TH and morphological differences cause differences in ∫CoT in adult zebrafish and whether these differences are temperature dependent. We conducted a fully factorial experiment where we manipulated acclimation temperature (24°C or 30°C) and thyroid status (control or hypothyroidism). We tested the hypotheses that (a) fish acclimated to 30°C have greater ∫CoT than fish acclimated to 24°C; (b) fish with induced hypothyroidism have higher ∫CoT than control fish, and that these effects are particularly pronounced at 24°C because of the role TH plays in cold acclimation of zebrafish (Little et al., 2013); (c) hypothyroid fish have reduced sustained swimming performance (*U*_crit_), tailbeat frequencies and tailbeat amplitudes because of reduced contractile capacity; and (d) increases in cross-sectional area and fineness ratios are positively and negatively associated with ∫CoT, respectively.

## MATERIALS AND METHODS

### Study animals and acclimation treatments

All experimental procedures were approved by the University of Sydney Animal Ethics Committee (approval number: 2021/1932). Adult zebrafish, *Danio rerio* (F. Hamilton, 1822), were obtained from a commercial supplier (Livefish, Bundaberg, QLD, Australia). For at least 1 week after arrival, fish were kept in large plastic tanks (0.6×0.4×0.35 m at a density of 1–2 fish per litre) in dechlorinated, filtered water at 27±0.5°C (maintained via submersible aquarium heaters; AquaOne, Kongs Pty Ltd, Ingleburn, NSW, Australia). Fish were fed daily to satiation with commercial flake fish food (TetraMin Tropical Flakes, Blacksburg, VA, USA), but fish were not fed for 24 h before experimental measurements were taken. The light cycle for all treatments was 14 h:10 h light:dark. The experiment was run in three blocks, and all treatments were represented in each block.

### Acclimation treatments

We conducted a factorial experiment with acclimation temperature (24°C or 30°C) and thyroid treatment (control or hypothyroid) as independent factors. Fish were randomly allocated to treatments and exposed to experimental conditions for 3 weeks. Fish were dispersed across three glass tanks (30×19×21 cm) per treatment each containing six fish (one tank per block). The temperature of the experimental tanks was regulated by placing the tanks into surrounding water baths (143.5×77.5×17.5 cm) which contained submersible heaters (200 W; AquaOne) and submersible pumps (5 W; SP-900, Resun, Shenzhen, China) to prevent temperature gradients.

Hypothyroidism was induced according to published protocols (Little et al., 2013) by maintaining tank water with 0.3 mmol l^−1^ propylthiouracil (Sapphire Bioscience, Redfern, NSW, Australia) dissolved in DMSO (0.05% v/v) to prevent the production of thyroxine (T_4_), and 5 μmol l^−1^ of iopanoic acid (Thermofisher Scientific Inc.) dissolved in DMSO (0.025% v/v) to inhibit deiodinase activity and thereby conversion of T_4_ to triiodothyronine (T_3_) and diiodothyronine (T_2_). Control tanks were maintained with equivalent amounts of DMSO to hypothyroid tanks. An 80% water change was conducted twice weekly to maintain water quality, and propylthiouracil, iopanoic acid and DMSO were replaced after water changes. We have previously (Little et al., 2013) validated the treatment effects in the same strain of zebrafish by measuring T_2_ and T_3_ in normothyroid and hypothyroid fish using LC-tandem mass spectrometry (Kunisue et al., 2010), demonstrating that the hypothyroid treatment did in fact substantially reduce or eliminate circulating T_2_ and T_3_ levels (tables 1 and 2 in Little et al., 2013). Additionally, we demonstrated that supplementation of hypothyroid fish with T_2_ and T_3_ raised their respective circulating levels. Supplementation with T_2_ and T_3_ also restored swimming performance to control levels, indicating that propylthiouracil and iopanoic acid per se do not affect performance (LeRoy and Seebacher, 2020; Little et al., 2013).

CoT was determined from oxygen consumption rates (*Ṁ*_O_2__) measured at different swimming speeds in a swimming flume respirometer (170 ml, Loligo Systems, Viborg, Denmark) according to published protocols (Jahn and Seebacher, 2019). Before measurements, fish rested inside the swimming flume for 90 min at a low flow speed (0.025 m s^−1^) to recover from handling stress (Seebacher et al., 2015). *Ṁ*_O_2__ was initially measured at a flow of 0.025 m s^−1^ and then at incrementally increased speeds of 0.1, 0.15, 0.2, 0.25, 0.3 and 0.35 m s^−1^. Measurements at a minimum of four speeds were necessary to calculate CoT from *Ṁ*_O_2__ measurements and we did not use data from fish that could not swim as fast as 0.2 m s^−1^, or data from individuals that swam unsteadily so that oxygen consumption measures were irregular; in total, we could not use data from one fish per treatment. CoT was measured at the acclimation temperature (24°C or 30°C; ±0.5°C) of each fish. The swimming flume was flushed with oxygenated water (via a submersible pump; Eheim, Deizisau, Germany) between measurements at different speeds to ensure full oxygenation at the start of each trial. The swimming flume and tank were drained, cleaned and dried daily to prevent build-up of microorganisms, and daily control trials were conducted in flumes without fish to determine possible contributions from microorganisms. Directly following CoT trials, we gently removed excess water from fish to measure body mass in a tared weighing boat containing just sufficient water to submerge fish.

### Swimming performance and kinematics

Critical swimming speed (*U*_crit_) was measured according to published protocols using Blazka-type swimming flumes (Seebacher et al., 2015). *U*_crit_ was measured at the acclimation temperature (24°C or 30°C; ±0.5°C) of each fish, and water temperature was maintained at the desired level with a submersible heater (100 W; AquaWorld, Australia) and monitored with a calibrated digital thermometer (Checktemp 4, Hanna Instruments, Woonsocket, RI, USA). We filmed fish from above while they were swimming steadily during *U*_crit_ trials (using a Hero 6 GoPro digital camera; GoPro Inc., San Mateo, CA, USA; filming at a resolution of 1080×720 pixels and 120 frames s^−1^) at two speeds (0.16 m s^−1^ and 0.28 m s^−1^) to determine tail beat amplitude (TBA) and tail beat frequency (TBF). TBA was determined as the maximal displacement of the tail at the peduncle during a single tail beat cycle. TBF (in Hz) was determined as 1/period, where period was the time taken for the tail to complete a single beat (Little et al., 2013). Swimming kinematics measures were averaged across ten independent tail beats when fish maintained their position against the flow and neither increased nor decreased their speed. Videos were analysed using Tracker Video Analysis software (version 5.1.5, Open Source Physics, www.opensourcephysics.org).

### Fineness ratio

Following swimming trials, fish were euthanised in a solution of ethyl 3-aminobenzoate methanesulphonate (MS-222; 0.3 g l^−1^ buffered to pH 7; Sigma-Aldrich), patted dry and weighed on an electronic balance (Satorius). Fish were then placed into a U-shaped sponge holder and the anterior side of the fish was photographed (with a Hero 6 GoPro digital camera, GoPro Inc.) to determine depth and cross-sectional area. Fish were then placed flat on their side and photographed to determine standard length. Cross-sectional area, maximum body depth and breadth were determined in ImageJ (Schneider et al., 2012). Fineness ratios was calculated as FR=(SL/$\sqrt{\mathrm{MDB}\times \mathrm{MBB}}$), where SL is the standard length (in cm), MBD is the maximum body depth (in cm), and MBB is the maximum body breadth (in cm) (Ohlberger et al., 2006).

Following experiments, one individual fish from the 30°C hypothyroid treatment was not included in the analysis because it was in poor condition [condition factor=0.9; average ±95% confidence interval (CI) for the treatment was 1.4±0.09] (McClelland et al., 2006). The responses we measured in this fish (∫CoT, *U*_crit_, TBA and TBF) were in the same direction as the average for that treatment. At the end of experiments, mean (±s.e.m.) fish mass and standard length were: 24°C, control 0.50±0.016 g, 3.30±0.040 cm (*N*=17); 24°C, hypothyroid 0.46±0.018 g, 3.18±0.060 cm (*N*=17); 30°C, control 0.49±0.027 g, 3.20±0.063 cm (*N*=17); 30°C, hypothyroid 0.45±0.029 g, 3.14±0.088 cm (*N*=16).

### Statistical analyses and calculations

*Ṁ*_O_2__ was extracted from the slope of the linear decrease in oxygen concentration at each speed, and CoT (in μmol kg^−1^ m^−1^) for each speed was calculated by dividing *Ṁ*_O_2__ (in μmol g^−1^ min^−1^) by swimming speed (in m s^−1^) (Claireaux et al., 2006). Power functions (*Y=aX^b^*) were fitted to the CoT data of individual fish (in GraphPad Prism version 9.0.2) and then integrated across all measured speeds to estimate the aerobic metabolic cost of movement across all speeds for each fish (integrated cost, ∫CoT, in W kg^−1^). The ∫CoT values allowed us to determine individual differences in the energetic cost of movement (Seebacher et al., 2015). CoT and ∫CoT incorporate oxygen consumption at rest as well as oxygen consumption associated with locomotion. To quantify the locomotor component alone (∫CoT_net_), resting oxygen consumption was estimated for each individual by fitting a power function to oxygen consumption measured across all swimming speeds. Power functions were extrapolated to a near-zero flow rate (0.0001 m s^−1^) to estimate resting oxygen consumption (Fig. S1), which was subtracted from total oxygen consumption to calculate ∫CoT_net_ (Claireaux et al., 2006).

All statistical analyses were conducted in R (version 4.3.3 ‘Angel Food Cake’) and R Studio (version 2021.09.0) using the ‘aovp’ function in package lmPerm 2.1.4 (https://github.com/mtorchiano/lmperm), or ‘aovperm’ in permuco 1.1.3 (Frossard and Renaud, 2021). Permutational methods were chosen as they have relaxed assumptions about the underlying distribution of data, and offer greater power when sample sizes are small relative to total population size (Drummond and Vowler, 2012). We report permutational *P*-values. We analysed the effects of fixed factors temperature (24°C and 30°C) and thyroid treatment (control or hypothyroid) on the dependent variables ∫CoT, ∫CoT_net_, *U*_crit_, cross-sectional area and fineness ratio. We conducted a regression analysis (using the lmp function in lmPerm) across the complete dataset with ∫CoT as a dependent variable and cross-sectional area and fineness as predictors to test the hypothesis that these morphological characteristics influence ∫CoT. TBA and TBF were analysed with the fixed factors temperature (24°C and 30°C), thyroid treatment (control or hypothyroid) and speed (0.16 and 0.28 m s^−1^), with individual ID used as a random factor to account for repeated measures of individuals at two speeds. As above, we conducted regression analyses to test whether altered swimming kinematics influenced ∫CoT. Length was included as a covariate in the analysis of *U*_crit_, cross-sectional area and TBA. Experimental block was not significant in any of the analyses. All treatments contained an even sex ratio, and we did not have any sex-specific hypotheses so we did not analyse sex explicitly. We calculated effect sizes as eta-squared (η^2^) in the R package effectsize 1.0.2 for all statistical analyses.

## RESULTS AND DISCUSSION

**Fig. 1.**
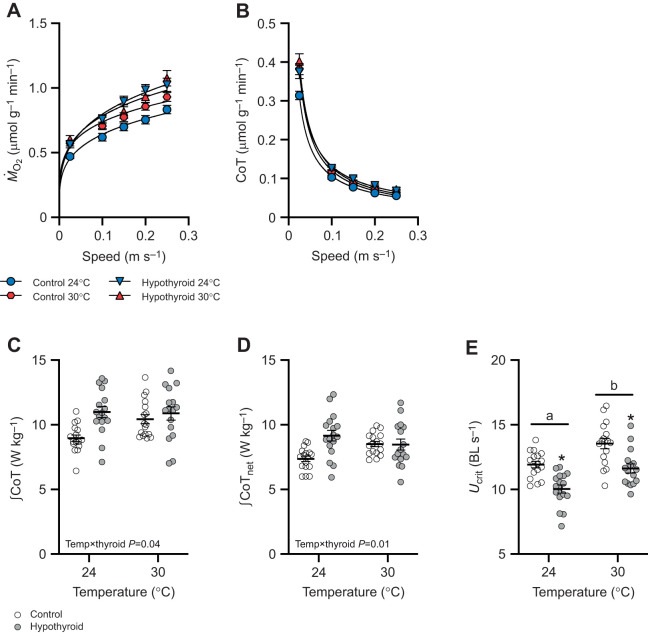
**Metabolic rate, cost of transport (CoT) and swimming speed.** (A) Metabolic rate (*Ṁ*_O_2__) of zebrafish increased with increasing swimming speed. The solid lines represent power functions fitted to the means from each treatment (24°C control: *y*=1.078*x*^0.23^, *R*^2^=0.99; 24°C hypothyroid: *y*=1.51*x*^0.28^, *R*^2^=0.98; 30°C control: *y*=1.13*x*^0.19^, *R*^2^=0.97; 30°C hypothyroid: *y*=1.24*x*^0.21^, *R*^2^=0.92). (B) CoT decreased with increasing swimming speed. The solid lines represent power curves fitted to the data from each treatment (24°C control: *y*=0.017*x*^−0.80^, *R*^2^=0.99; 24°C hypothyroid: *y*=0.023*x*^−0.76^, *R*^2^=0.99; 30°C control: *y*=0.019*x*^−0.81^, *R*^2^=0.99; 30°C hypothyroid: *y*=0.019*x*^−0.83^, *R*^2^=0.99). (C) Integrated CoT over speed (∫CoT) increased with hypothyroidism (*P*=0.0012), and there was a significant interaction between temperature and thyroid treatment (*P*=0.037). (D) Similarly, net integrated CoT (∫CoT_net_) was significantly higher at the warmer temperature, and there was a significant interaction between temperature and thyroid treatment (*P*=0.014). (E) Temperature (*P*<0.001) and thyroid treatment (*P*<0.001) had significant main effects on critical swimming speed (*U*_crit_), but there was no significant interaction between the two factors (*P*=0.96). BL, body lengths. Bars and symbols show means±s.e.m., *N*=16–17 fish per treatment; significant differences between treatments are indicated by asterisks, and between temperatures by different letters (permutational analysis of variance).

Our results show that thyroid hormone influences the energetics of movement in animals: ∫CoT was greater in 30°C- compared with 24°C-acclimated fish (hypothesis a) and hypothyroid fish had higher ∫CoT (hypothesis b). Metabolic rate increased with increasing swimming speed (Fig. 1A) and CoT decreased with increasing swimming speed (Fig. 1B). Both ∫CoT and ∫CoT_net_ increased significantly with hypothyroidism, but there was no significant main effect of temperature; however there was a significant interaction between the two factors, indicating that the effect of hypothyroidism was greater in 24°C-acclimated fish (Table S1; Fig. 1C,D). The parallel responses of ∫CoT and ∫CoT_net_ show that differences in ∫CoT were not driven by variation in resting metabolic rate but by the energetic cost of movement per se. Hypothyroidism reduced swimming speed (hypothesis c). *U*_crit_ was significantly greater in fish acclimated to 30°C than in those acclimated to 24°C, and it was significantly greater in control fish compared with those from the hypothyroid treatment (main effects; Table S1); there was no significant interaction between the two factors (Table S1; Fig. 1E). These data indicate that the action of thyroid hormone reduces the energetic cost of swimming while increasing maximal performance.

**Fig. 2.**
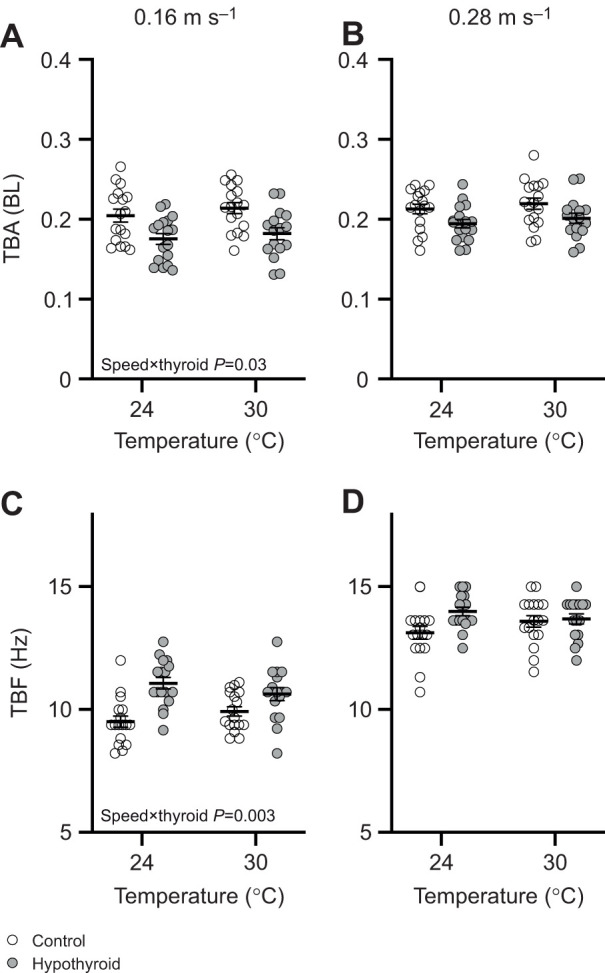
**Swimming kinematics.** Speed and thyroid treatment, but not temperature (*P*=0.64), had significant effects on tail beat amplitude (TBA; both *P*<0.001; A,B) and frequency (TBF; both *P*<0.001; C,D) at 0.16 m s^−1^ (left) and 0.28 m s^−1^ (right). There were significant interactions between speed and thyroid treatment in their effects on TBA (*P*=0.029) and TBF (*P*=0.0028; indicated in panels). Bars and symbols show means±s.e.m., *N*=16–17 fish per treatment.

The TH-mediated differences in swimming performance were associated with changes in swimming kinematics. TBA was significantly greater at 0.28 m s^−1^ than at 0.16 m s^−1^, and it was significantly greater in control fish compared with hypothyroid fish (main effects; Table S2). There was no significant main effect of acclimation temperature (Table S2; Fig. 2A,B). There was a significant interaction between speed and thyroid treatment (Table S2; Fig. 2A,B), indicating that the effect of hypothyroidism was somewhat greater at 0.16 m s^−1^. The interactions between speed and temperature, and temperature and thyroid treatment were not significant, nor was the three-way interaction (Table S2; Fig. 2A,B).

TBF was significantly greater in fish swimming at 0.28 m s^−1^ compared with 0.16 m s^−1^, and there was a main effect of thyroid treatment but not temperature (Table S2; Fig. 2C,D). Similar to TBA, there was a significant interaction between speed and thyroid treatment (Table S2; Fig. 2C,D), and the interactions between speed and temperature, and temperature and thyroid treatment were not significant, nor was the three-way interaction (Table S2; Fig. 2C,D).

Neither TBA (0.16 m s^−1^: *R*^2^=−0.01, *P*=0.58; 0.28 m s^−1^: *R*^2^=−0.003, *P*=0.62) nor TBF (0.16 m s^−1^: *R*^2^=−0.01, *P*=0.78; 0.28 m s^−1^: *R*^2^=0.004, *P*=0.26) were correlated significantly with ∫CoT (Fig. S2A,B). The differences in swimming kinematics between the control and hypothyroid treatments provide functional insight into variation in muscle contractile dynamics. Locomotor performance depends on muscle calcium cycling dynamics (Gundersen, 2011) and ATP supply for crossbridge formation and SERCA activity (Hargreaves and Spriet, 2020). TH increases myosin and SERCA activity, which can enhance locomotion by enhancing muscle contraction and relaxation dynamics (Salvatore et al., 2014). Hypothyroidism reduces maximal SERCA activity in the skeletal muscle of zebrafish, particularly in cold-acclimated fish (Little and Seebacher, 2013). These decreases are paralleled by decreased metabolic rate and heart rate (Little, 2021), leading to an overall less oxidatively poised phenotype. Increased ∫CoT in exercising hypothyroid fish may reflect this shift towards less oxidative muscle, which may be more reliant on the metabolically less efficient fast muscle (type II) fibres to sustain continuous movement.

Earlier recruitment of type II fibres would increase ATP expenditure at lower speeds and limit the capacity to further increase relaxation rate as speed increases. This mechanism is consistent with the increased TBF seen in hypothyroid fish at lower speeds and the reduced ability to modulate TBF with increasing speed. The higher TBA in control fish at both speeds suggests improved propulsive force generation, although changes in amplitude are not tightly correlated with changes in speed (Hawkins et al., 2025). In contrast, hypothyroid fish exhibited altered frequency–amplitude combinations that deviated from more efficient patterns seen in control fish. Control fish primarily increased speed through TBF modulation rather than TBA, a strategy associated with increased efficiency (Li et al., 2021) and lower ∫CoT in our study. We note, however, that our data leave the exact mechanisms unresolved and the lack of correlation between ∫CoT and TBA and TBF indicates that there is no direct influence of tail beat kinematics on ∫CoT. It is likely that ∫CoT is determined by a range of different mechanisms as briefly outlined above, and that tail beat kinematics may influence ∫CoT indirectly via shared pathways that are modulated by TH signalling.

**Fig. 3.**
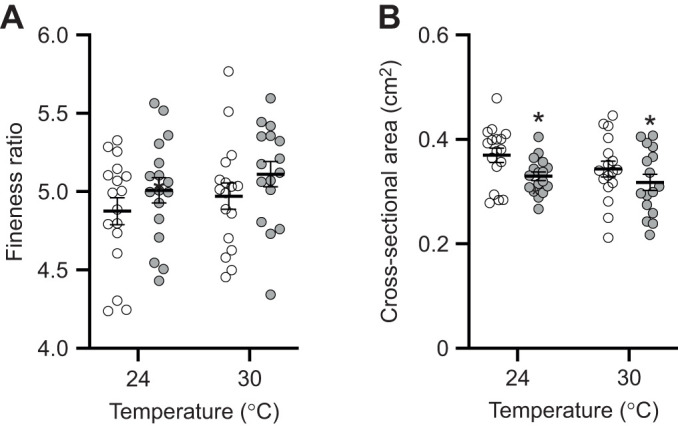
**Morphology.** (A) There was no significant main effect of either temperature or thyroid treatment on fineness ratio, nor was there a significant interaction between the two factors (all *P*>0.1). (B) Hypothyroidism significantly decreased cross-sectional area (*P*=0.0066), but there was no significant effect of temperature and no interaction between the two factors (all *P*>0.1). Bars and symbols show means±s.e.m., *N*=16–17 fish per treatment; significant differences between treatments are shown by asterisks (permutational analysis of variance).

Finally, cross-sectional area and fineness ratios were not associated with ∫CoT (hypothesis d). Neither acclimation temperature nor thyroid treatment significantly influenced fineness ratio, nor was there a significant interaction between the two main factors (Table S1; Fig. 3A). Cross-sectional area was significantly greater in control fish compared with hypothyroid fish, which may be associated with a reduction in motor units (Salvatore et al., 2014). There was no significant difference between acclimation temperatures, nor was there a significant interaction between the two factors (Table S1; Fig. 3B). Neither cross-sectional area (*R*^2^=0.07, *P*=0.20) nor fineness ratio (*R*^2^=−0.005, *P*=0.51) influenced ∫CoT significantly (Fig. S1C,D). These data indicate that differences in morphology between zebrafish were not great enough to influence swimming performance and energetics, and that morphology is important only in determining differences in swimming at higher taxonomic levels such as between species (Liao, 2022).

Together, our results suggest that TH improves swimming performance by supporting optimal tailbeat kinematics and reducing the energetic costs of sustained locomotion, particularly under cooler thermal conditions. The reduced effects of TH at warmer temperatures indicate that as global temperatures rise, the capacity for TH signalling to regulate movement performance in ectotherms may become compromised (Zwahlen et al., 2024). Increased temperatures reduced movement efficiency but increased movement capacity, so that loss of efficiency may be the primary constraint on ectotherm locomotion under climate change. If food availability and processing capacity do not increase proportionally with energetic costs, there may be energy deficits that reduce movement capacity and exacerbate potential trade-offs with other energy-demanding traits, such as maintenance and reproduction (Pontzer and McGrosky, 2022). We acknowledge that we used a laboratory-strain zebrafish model so that extrapolations to wild populations must be treated with caution. Nonetheless, our data indicate that it is important to confirm the validity of our findings for natural populations because of the potentially far-reaching ecological implications. Additionally, human activity has dramatically increased endocrine-disrupting chemicals derived from plastics (e.g. bisphenols) in aquatic environments globally, which have pronounced effects on TH signalling (Li et al., 2026) and can thereby affect ∫CoT and energetics of organisms. The action of bisphenols is temperature dependent (Wu and Seebacher, 2021) so that a natural thermal gradient and temperature change can modify the energetics of movement and its ecological consequences. Plastic pollution and climate change are two of the most pressing global crises (Einhäupl et al., 2026), and their effects on ecosystems are mediated at least partly by impacting the physiology of individuals, as our data exemplify. Physiological measurements must therefore be incorporated into conservation to achieve the most effective outcomes.

## Supplementary Material

10.1242/jexbio.252605_sup1 Supplementary information
