# Supplementary material for: Thyroid hormone determines energy efficiency of locomotion in zebrafish (Danio rerio) in a temperature-sensitive manner
Source: J Exp Biol. 2026 Jul 16;229(14):jeb252605. doi: 10.1242/jeb.252605 (PMC13405219; doi:10.1242/jeb.252605)
Supplement: Supplementary information [file jexbio-229-252605-s1.pdf]

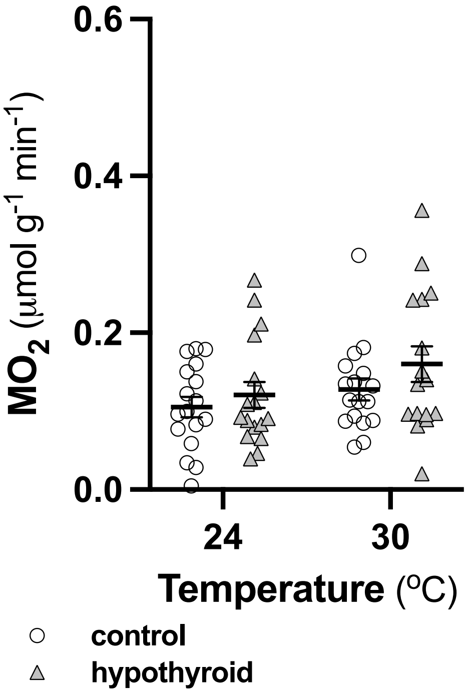

Fig. S1. Resting oxygen consumption estimated from swimming trials. Horizontal bars represent means ± s.e. and symbols represent data from individual fish (open circles = control treatment, filled circles = hypothyroid treatment).

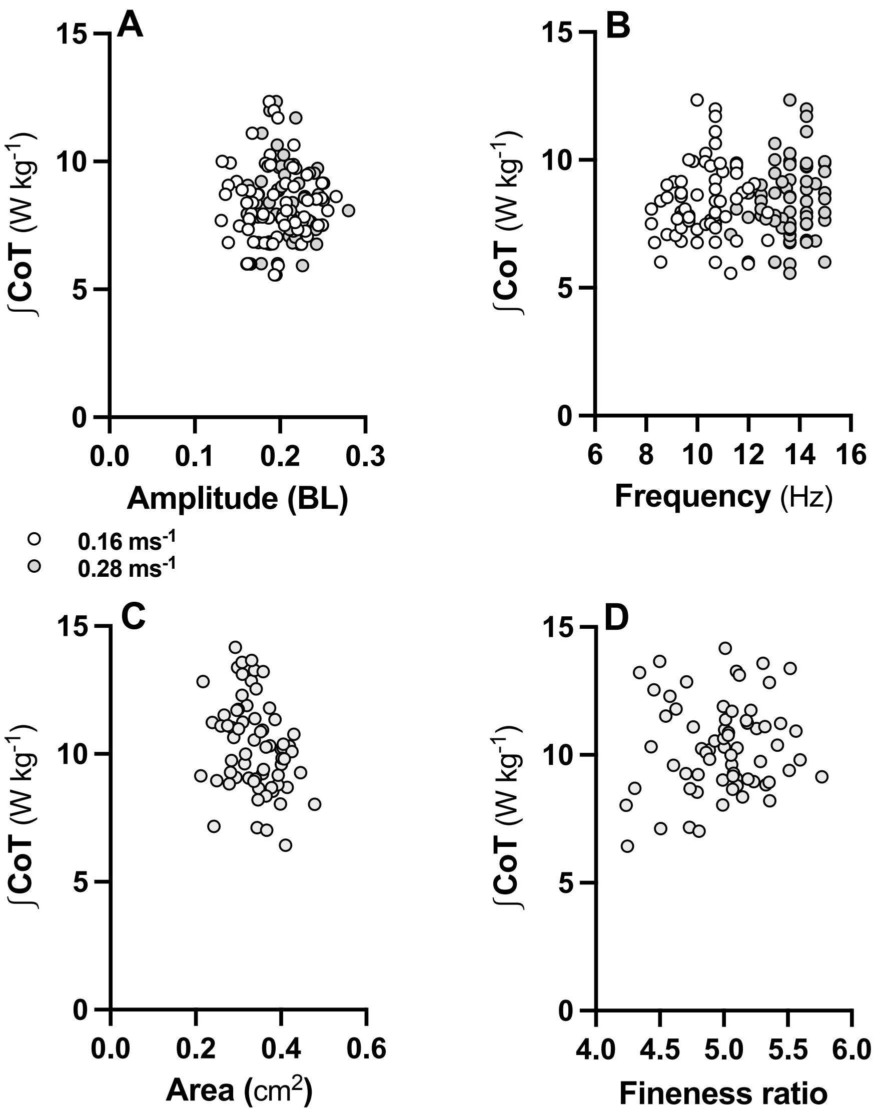

Fig. S2. Relationship between ∫CoT and morphology and swimming kinematics. There was no significant association between ∫CoT and tail beat amplitude (A), tail beat frequency (B), cross-sectional area (C), or fineness ratio (D). Data from individual fish across all treatments are shown.

Table S1. Results of permutational analyses. We tested the effects of the main factors temperature (T) and thyroid treatment (Trt) on integrated cost of transport (∫CoT), net integrated cost of transport (∫CoT_{net}), swimming performance (U_{crit}), cross sectional area, and fineness ratio; permutational *p*-values are shown. For ∫CoT, ∫CoT_{net}, and fineness ratio df = 1, 61, and for U_{crit} and cross-sectional area df = 1, 60. Significant results when $p < 0.05$ are highlighted in bold, and effect sizes are shown as eta-squared (η^2).

Trait	T	η^2	Trt	η^2	T*Trt	η^2
∫CoT	0.10	0.05	**0.0012**	0.13	**0.037**	0.06
∫CoT_{net}	0.66	<0.01	**0.0046**	0.10	**0.014**	0.11
U_{crit}	**< 0.001**	0.27	**< 0.001**	0.34	0.96	<0.01
Fineness ratio	0.26	0.02	0.13	0.04	0.98	<0.01
Cross-sectional area	0.11	0.03	**0.0066**	0.09	0.98	<0.01

Table S2. Results of permutational analyses. We tested the effects of the independent factors speed (S), temperature (T), and thyroid treatment (Trt) on tailbeat amplitude and tailbeat frequency; permutational *p*-values are shown. df = 1, 63 for both analyses. Significant effects ($p < 0.05$) are shown in bold, and effect sizes are shown as eta-squared (η^2).

Factor	Tailbeat amplitude	$\eta^2_{amplitude}$	Tailbeat frequency	$\eta^2_{frequency}$
S	**< 0.001**	0.05	**< 0.001**	0.77
T	0.64	<0.01	0.87	<0.01
Trt	**< 0.001**	0.23	**<0.001**	0.17
S*T	0.85	<0.01	0.64	<0.01
S*Trt	**0.029**	0.01	**0.0028**	0.03
T*Trt	0.90	<0.01	0.053	0.05
S*T*Trt	0.84	<0.01	0.82	<0.01
